# Identification of RNA Editing Sites Reveals Functional Modifications with the Addition of Methionine to the Daily Rations of Yaks

**DOI:** 10.3390/ani15020171

**Published:** 2025-01-10

**Authors:** Shiyu Wu, Xinrui Liu, Yaxin Liu, Shikai Wang, Wei Peng, Ming Zhang, Binglin Yue, Hui Wang, Jikun Wang, Jincheng Zhong, Fang Sun, Yixi Kangzhu, Jiabo Wang

**Affiliations:** 1 Key Laboratory of Qinghai-Tibetan Plateau Animal Genetic Resource Reservation and Utilization, Ministry of Education and Sichuan Province, Southwest Minzu University, Chengdu 610225, Chinazhongjincheng518@163.com (J.Z.); 2 Qinghai Academy of Animal Science and Veterinary Science, Qinghai University, Xining 810016, China; 3 Key Laboratory of Combining Farming and Animal Husbandry, Ministry of Agriculture, Institute of Animal-Husbandry, Heilongjiang Academy of Agricultural Sciences, Harbin 150028, China; hljxmsf@163.com

**Keywords:** *Bos grunniens*, RNA-seq, fatten, JACUSA, SPRINT, REDItools

## Abstract

Methionine is an amino acid necessary for the growth and development of all animals. Glutathione produced during methionine metabolism can reduce damage to cells caused by oxidative stress. Supplementing restricted amino acids in animals by scientific means will be beneficial to protein synthesis, which will affect the growth and development of animals and will bring huge economic benefits when applied to actual production and life. In this study, we collected three muscle tissues from 24 male Maiwa yaks, which were fattened for three months with different methionine concentrations in their diet. RNA-seq was performed to obtain expression reads. A total of 1116 editing sites were identified by at least two software; the editing site types were mainly T-to-C and A-to-G mutations. We found two significant RNA editing sites presenting high-risk editing types. One was located on the *MSRA* gene that regulates the reduction of methionine, and the other can make changes to the properties of encoded proteins. This provides further understanding of the mechanism of yak muscle tissue and regulation of gene expression after the addition of methionine to daily rations.

## Simple Summary

Methionine is an essential amino acid for animal growth and development. In this study, we added four different doses of methionine to the yak basal diet to explore the impact of adding methionine on RNA editing sites in yak muscle tissue. After filtering, we detected a total of 1116 RNA editing sites, the main types of which are T-to-C and A-to-G. Most of them were enriched in muscle development pathways, such as extracellular matrix (ECM) interactions and thyroid hormone synthesis. The two significant RNA editing sites present high-risk editing types. This provides further understanding of the mechanism of yak muscle tissue and regulation of gene expression after the addition of methionine to daily rations.

## 1. Introduction

Methionine (Met) can improve protein and fat deposition in animals and affect muscle cell development. It is an essential amino acid in mammals [1] and acts in many important metabolic and non-metabolic pathways. Met can serve as a precursor of carnitine, creatine, cysteine, homocysteine, and succinyl-CoA [2]. In addition, met also acts on lipid metabolism and the activation of antioxidant enzymes, such as methionine sulfoxide reductase A and glutathione biosynthesis, which are important molecules for anti-oxidative stress [3]. Some studies have found that adding DL-methionine (Dextrogyre and levogyre-isomers) and methionine hydroxy analogs to low-basal diets can increase RNA expression levels in muscles, improve protein synthesis efficiency, and promote muscle growth [4]. These pathophysiological events may lead to lipid accumulation in the liver, thereby triggering the development of non-alcoholic fatty liver disease [5].

Yak (*Bos grunniens*) is a rare ruminant species indigenous to the Qinghai-Tibet Plateau and surrounding regions [6]. It can adapt to harsh alpine temperatures at 3000 m above sea level and utilize grassland resources for subsistence. The Maiwa yak is a notable breed of the northwest Sichuan Plateau. It was discovered in the early 1980s during China’s National Livestock and Poultry Resource Survey [7]. However, because of the harsh living environment of yaks and as yaks rely only on natural pastures for nutrition in the traditional grazing model, their access to nutrients such as methionine is low. Methionine is the most limiting amino acid in ruminant growth [8]. A lack of methionine in feed can cause many adverse effects, including toxemia, muscle paralysis, damp heat, depression, alopecia, schizophrenia, liver disease, and changes in growth performance [9]. Genetic defects in human methionine metabolism can lead to cystathionine symptoms such as etheruria, homocystinuria, and hyperthioninemia. Correspondingly, excessive methionine is toxic to animals, inhibiting their growth and development, decreasing production performance, and, in severe cases, causing death [9]. Adding 18 g of methionine to the diet of 7-week-old Holstein cows leads to weight loss and reduced nitrogen utilization [10]. Adding RP-Met to the compound chemically treated rice straw diet can improve the daily weight gain, net meat rate and meat quality of barn-fed Tan sheep to a certain extent [11]. This shows methionine’s key role in growth and development and the importance of controlling its levels in animal feed. Adding methionine to the basal diet can improve the production performance of cattle. Fundamental research conducted by Richardson [12] indicates that the order of the three limiting amino acids in growing cattle is Met, Lys, and Thr. Therefore, Met is the most valuable limiting amino acid for research on growing and fattening cattle. Under the addition of Methionine to daily rations of the yak diet, the response of the organism should be caused by some gene-level variations.

RNA editing sites (REs) refer to specific sites where nucleotide sequence changes occur at the RNA level, and they are widely present in nature. Edits at these sites modify the expression of key genes. The process of pyrimidine exchange in RNA transcripts, called RNA editing, can change the nucleotide composition of a transcript through the insertion, deletion, and substitution of nucleotides [13]. In 1986, RNA editing was first discovered in trypanosomatid mitochondria, and it has since been discovered in nuclei, chloroplasts, and plasmids [14]. RNA editing is common in mammals. Three types of RNA editing are known to exist in humans [15], chickens [16], sheep [17], pigs [18], and cattle [19]. In human and mammal genetic research, most of the A-to-I (A-to-G) and T-to-C REs were reported, which were catalyzed by adenosine deaminase acting on the RNA (ADAR) family of enzymes [20]. The ADAR enzymes bind double-stranded RNAs and deaminate adenosine to inosine, which is recognized as guanosine by the cellular machinery. A-to-I editing is pervasive in Alu repeats (a type of short interspersed nuclear elements (SINEs), which are repetitive DNA sequences scattered throughout the genome of primates, including humans) because of the double-stranded RNA structures formed by inverted Alu repeats in many genes. RNA editing can be affected by the external environment and, thus, change. Zhang [21] and Li [22] proved that REs are one of the main responses of gene expression in animals and plants under environmental changes. In this study, we observed the variation in RNA levels (RNA editing sites) based on the genome level. Through the generalized locations, functions, and risk levels of these RNA editing sites, we aim to figure out the key functional genes and response mechanisms in yak muscle by the addition of methionine.

## 2. Materials and Methods

### 2.1. Data Resource

Twenty-four Maiwa male yaks, approximately four years old, with similar weights (252.79 ± 15.95 kg) and health, from a breeding farm at an altitude of approximately 2500 m in Xiaojin County, Aba Qiang Autonomous Region, Sichuan Province, China, were obtained for this study. These animals were divided equally into four groups (six yaks in each group) for experimentation. Yaks were fed, according to their group, for three months with no rumen, protected methionine (RP-Met) (0 g/d), a small amount of RP-Met (5 g/d), a medium amount of RP-Met (10 g/d), and a large amount of RP-Met (15 g/d). RP-Met was added to the basal diet (Table S1). The animals were fed twice daily (7:30 and 15:30) in single stalls, which complied with animal welfare guidelines. The nutritional composition of feed is shown in Table S2. All animals were slaughtered in an orderly manner at similar times on the same day. All tissues were collected rapidly after slaughter. After slaughter, small pieces (approximately 1 cm^3^ each) of the longissimus dorsi (middle), buttock (surface), and pectoralis (front end) muscles were collected and then stored in liquid nitrogen for subsequent experiments and analyses. Fat collection was avoided during sampling.

### 2.2. RNA-Seq Sequencing

RNA libraries were established for individual yaks. Total RNA was extracted from the specimens using a TRIzol^®^ Plus RNA Purification Kit (Invitrogen, San Diego, CA, USA). The Ribosomal RNA (rRNA) was eliminated before sequencing using the Epicenter Ribo-zeroTM rRNA Removal Kit (Illumina, Beijing Genomics institution, Shenzhen, China), according to the manufacturer’s instructions. The cDNA sequence from total RNA was obtained through reverse transcription, followed by fragmentation, PCR amplification, and high-throughput sequencing using the Illumina HiSeq 4000 sequencing platform. All RIN values of samples were above 9.2, which indicates the quality of our RNA extraction was suitable.

### 2.3. Quality Control and Read Mapping

We obtained Fastq format sequencing files for the total RNA from the longissimus dorsi, buttock, and pectoralis muscles. First, we used FastQC [23] (v0.11.9) software to check the raw RNA-seq reads, and then we used Fastp [24] (v0.23.4) software to perform quality control operations according to default parameters. We used HISAT2 (2.21) [25] software to map the clean reads of the RNA-seq data with a yak reference genome sequence (Bosgru_v3.0) on Ensemble and generated a SAM file. Samtools (version 1.18) [26] were used to convert them to BAM files.

### 2.4. RNA Editing Sites Detection

JACUSA(1.2.0) [27], SPRINT(0.1.8) [28], and REDItools(1.2.0) software [29] were used to identify editing sites using default parameters. REDItools [29] detects RNA editing sites from BAM files and includes filtering, annotation, and analysis tools but has a slow runtime. JACUSA [27] compares RNA-seq data with biological repeats to quickly and accurately detect single nucleotide variations. SPRINT [28] can identify RNA editing sites without SNP filtering and detect super editing sites from remapped reads, automating RNA-seq analysis. In SPRINT [28], we used the sprint_from_bam command to operate BAM files. We obtained predicted RNA editing sites from the 24 individual yaks across three tissues and four groups. A self-written R script was used to filter RNA editing sites in the same tissues for all four groups. We used the intersection between files of the same muscle tissue in each group and the union of the four groups of the same muscle tissue after the intersection to obtain RNA editing sites in the three muscle tissues. The VennDiagram [30] package (1.7.3) in R (version 4.3.2) was used to create a Venn diagram of intersecting RNA editing sites in the three types of muscle tissues after filtering and selected those detected by at least two tools as candidate REs for further analysis.

### 2.5. Prediction of High-Risk Variant Sites

The HaplotypeCaller function of gatk4.0 [31] was used to convert the BAM files to GVCF files. GenotypeGVCFs and CombineGVCFs were used to merge the GVCF files, which were then referred to as nucleotide mutations (VCF file). The detected RNA editing sites in the three tissues were used to match the nucleotide mutation VCF file. SnpEff (4.3.1) [32] software was used to annotate the filtered VCF file and predict the risk level of each RE. Risk levels were classified as high, moderate, low, or modifiers. High risk indicates that the variant is expected to have a significant (disruptive) effect on the protein, like protein truncation, loss of function, or nonsense-mediated decay; moderate risk indicates a non-disruptive variant that might change protein effectiveness; low risk indicates a variant assumed to be mostly harmless or unlikely to change protein behavior; modifier indicates non-coding variants or variants affecting non-coding genes, where predictions are uncertain or lack evidence of impact [32].

### 2.6. Annotation and Enrichment Analysis

We extracted the REs in the functional genes and performed gene ontology (GO) and Kyoto Encyclopedia of Genes and Genomes (KEGG) analyses using the R language. The GO function enrichment annotation file for org.Bt.eg.db was obtained using the AnnotationHub::AnnotationHub() function in the R package AnnotationHub (3.10.0) [33]. The function enrichGO() of the R package clusterProfiler (4.10.1) [34] was used to perform the GO analysis; for the KEGG analysis, we used the function mapIds() of the R package AnnotationDbi (1.68) [35] to obtain the Entrez ID corresponding to the gene in the NCBI database. The enrichKEGG() function of the R package clusterProfiler (4.10.1) [32] was used to enrich the set analysis. We opted to display the top 15 enriched items and pathways and set the effect to be significant at *p* < 0.05.

## 3. Results

### 3.1. RNA Sequencing and the Distribution of RNA Editing Sites

**Figure 1:**
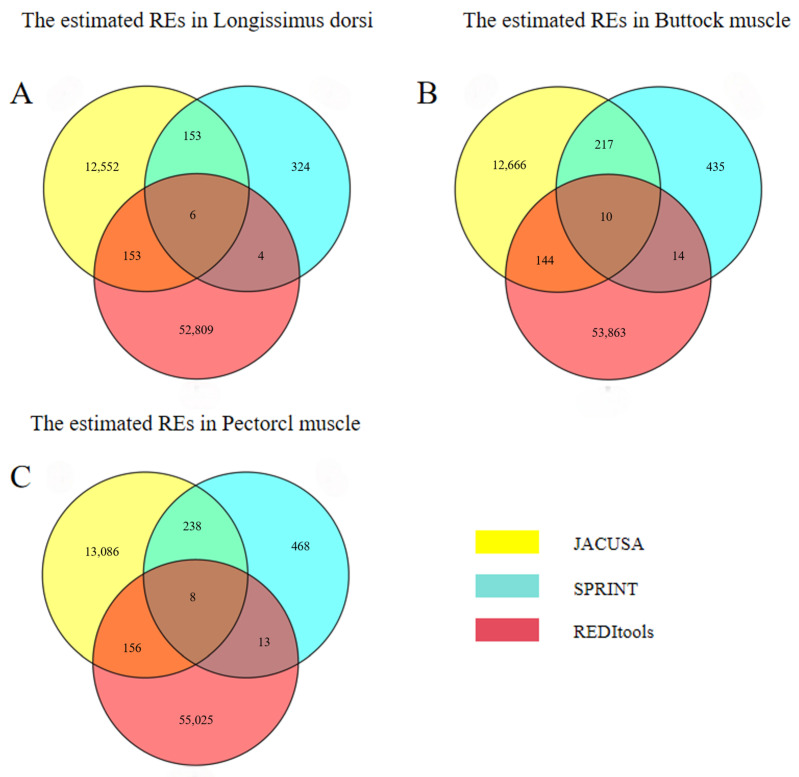
The Venn plots of REs predicted among JACUSA, SPRINT, and REDItools. Based on the position information of predicted REs in the yak genome. Three software, including JACUSA (yellow), SPRINT (blue), and REDITOOLS (red), were used to detect RNA editing sites in longissimus dorsi (**A**), buttock muscle (**B**), and pectoral muscle (**C**) tissues.

We obtained 2,314,821,308 total clean reads for the longissimus dorsi, 2,254,137,032 for the buttock muscle, and 2,285,468,019 for the pectoral muscle. The overall mapping ratio of reads to the reference genome across all samples ranged from 94.55 to 94.94% (Table S3). We obtained 12,864, 13,037, and 13,488 REs by JACUSA [27] in the longissimus dorsi, buttock, and pectoral muscles, 487, 676, and 727 REs by SPRINT [28], and 52,972, 54,031, and 55,202 REs by REDItools [29] (Table S4). The REs detected by at least two software were considered as candidate REs for further analysis. Finally, there were 316 in the longissimus dorsi, 385 in the buttock muscle, and 415 candidate sites in the pectoral muscle (Figure 1).

**Figure 2:**
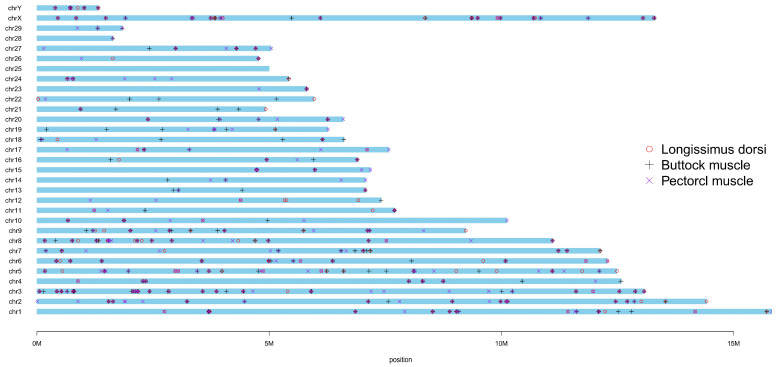
The distribution of RNA editing sites throughout the yak genome. Distribution map of REs detected by at least two of the three software on the chromosomes. The relative positions of detected REs in the yak genome were labeled in 29 autosomes and 2 sex chromosomes. The histogram length represents the chromosome length, and three symbols represent the three muscle tissues.

**Figure 3:**
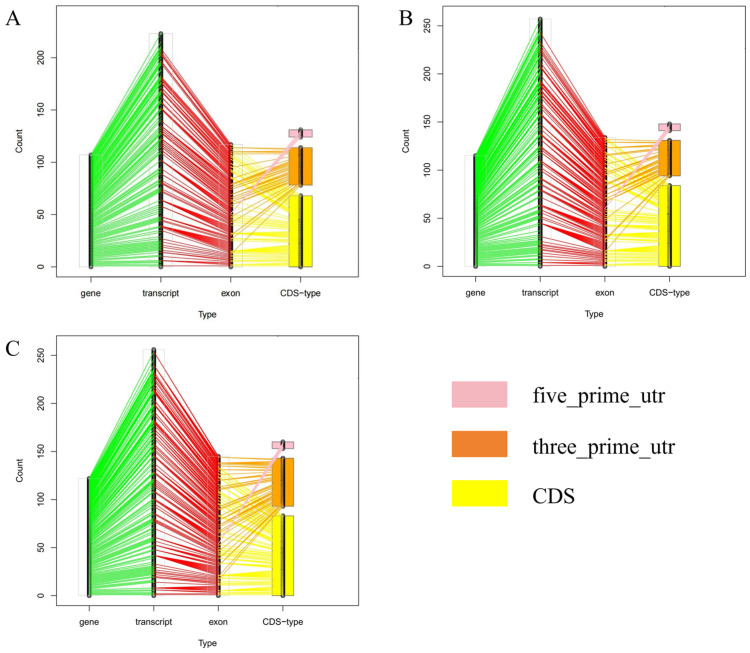
The relative position of the REs types in the whole genome. All detected REs of the three muscle tissues, including the longissimus dorsi (**A**), buttock muscle (**B**), and pectoral muscle (**C**), were matched to the position of the yak reference genome. The CDS type includes five_prime_utr, three_prime_utr, and CDS, and the REs are classified one by one according to the gene_id.

The RNA editing sites showed a non-uniform distribution across the yak chromosomes (Figure 2). The most editing sites (130) were observed on chromosome 3, while the fewest editing sites (2) were found on chromosome 28. Among the detected editing sites, T-to-C and A-to-G edits were the most common, accounting for more than 25%, and unique G-to-C/G-to-T and C-to-G/C-to-T edits were detected in the longissimus dorsi and buttocks (Table S5). We compared the RNA editing sites with the annotation files of the yak reference genome to obtain the RNA types corresponding to these sites (Figure 3). The RNA editing sites in the muscle tissues were located in the gene, transcript, exon, coding sequence types, and CDS-type sections, including five_prime_utr, three_prime_utr, and coding sequences sections, all of which showed correct correspondences. We also found that the five_prime_utr genes in the three tissues corresponded to the same exon: methionine sulfoxide reductase A (*MSRA*), which encodes a ubiquitous and highly conserved protein that catalyzes the enzymatic reduction in methionine sulfoxide to methionine.

### 3.2. Risk Prediction

**Figure 4:**
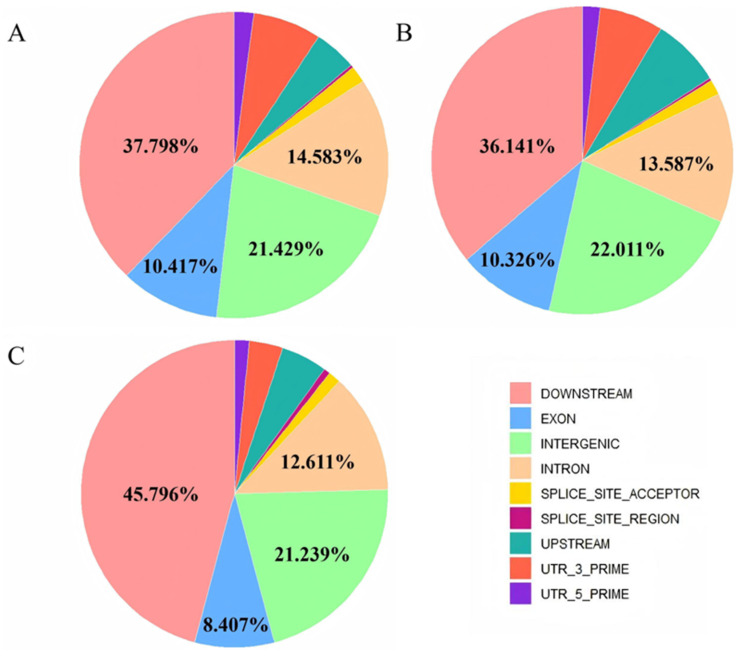
The proportion of identified REs types throughout the genome.All detected REs of the three muscle tissues, including the longissimus dorsi (**A**), buttock muscle (**B**), and pectoral muscle (**C**),were compared. The types and proportions of genomic intervals were identified and visualized using SnpEff(4.3.1) [32] software and the ggplot2(3.5.0) package in the R language. The most common types of the three tissues are DOWNSTREAM, EXON, INTERGENIC, and INTRON.

RNA editing site impacts were predicted by SnpEff [32]. A total of 1116 impacted sites were detected in the three tissues, with 12 high-impact sites in the longissimus dorsi and pectoralis muscles and 13 high-impact sites in the buttock muscle (Table S6). Longissimus dorsi and buttock muscles showed the most mutation sites on chromosome 3, and pectoral muscles showed the most mutation sites on chromosome X, with most variants being single-nucleotide polymorphisms (SNPs). Nearly 70% of the editing sites are missense mutations, and the rest are silent mutations (Figure 4). The main types of mutations were intergenic, and they were concentrated mostly in the downstream region of genes. The high-risk genes common to the three muscle tissues were *CD36*, *GPKOW*, *HNRNPLL*, *SMS*, and *TMOD4. CD36* and *TMOD4* are involved in muscle development. *CD36* is involved in muscle lipid utilization, *TMOD4* is involved in actin- and tropomyosin-binding processes, and *HNRNPLL* is related to translation control.

### 3.3. KEGG and GO Analyses

**Figure 5:**
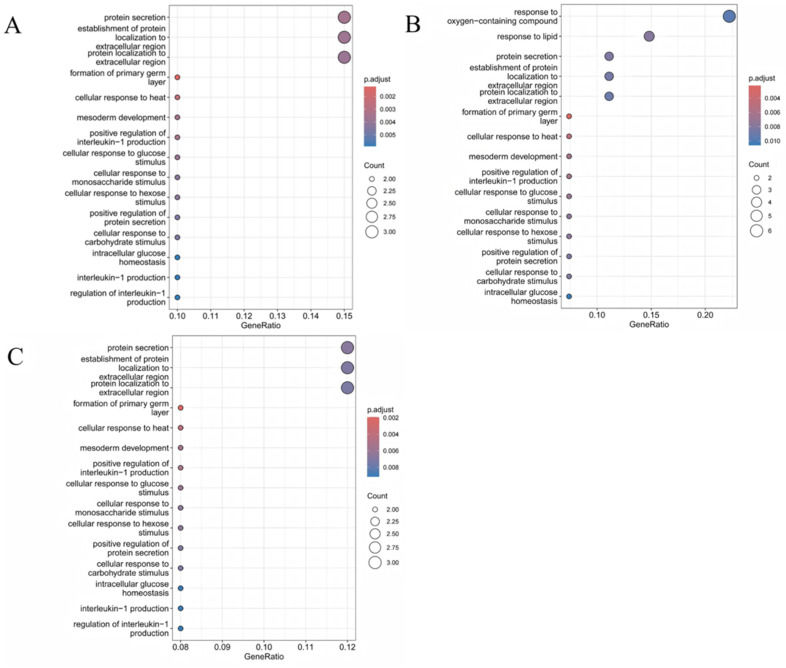
The GO analysis of functional genes in 3 tissues. The GO analysis was performed to locate the functional gene family using the AnnotationHub package in the R language. The 3 tissues, including the the longissimus dorsi (**A**), the buttock muscle (**B**), and the pectoral muscle (**C**), were compared. The size of the circle is proportional to the number of genes. The top 15 significant GO terms are labeled in the figure. Color was used to present significant levels.

**Figure 6:**
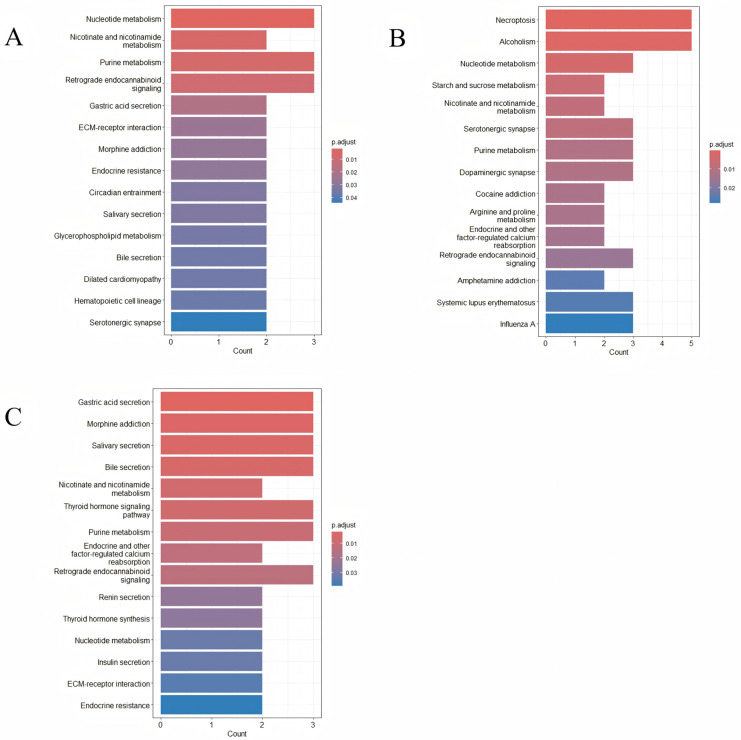
The KEGG analysis of functional genes in 3 tissues. The KEGG analysis was performed to locate functional pathways using the clusterProfiler package in the R language. The 3 tissues, including the longissimus dorsi (**A**), the buttock muscle (**B**), and the pectoral muscle (**C**), were compared. The top 15 significant KEGG pathways are labeled in the figure. Color was used to present significant levels.

GO (Figure 5) and KEGG (Figure 6) pathway enrichment analyses were performed to understand the functional roles of these target genes. The number of muscles used for analysis in the three muscle groups was 64, 75, and 87. In the longissimus dorsi, there are 572 biological process (BP), 111 cellular component (CC), 73 molecular function (MF) terms, and the corresponding numbers in the buttock and pectoral muscles are 688, 130, and 94, and 680, 122, and 75. All entries presented in the figure belong to the BP; the top five terms with the most enriched genes were protein secretion, establishment of protein localization to the extracellular region, protein localization to the extracellular region, response to oxygen-containing compounds, and response to lipids. According to KEGG analyses, the affected target genes were enriched in pathways such as nucleotide metabolism, purine metabolism, glycerophospholipid metabolism, extracellular matrix (ECM) interactions, thyroid hormone synthesis, and insulin secretion. The high-risk gene *CD36* was enriched in the ECM–receptor pathway, which regulates the proliferation of preadipocytes (*p*-values are in Table S7).

### 3.4. REs Verification

**Figure 7:**
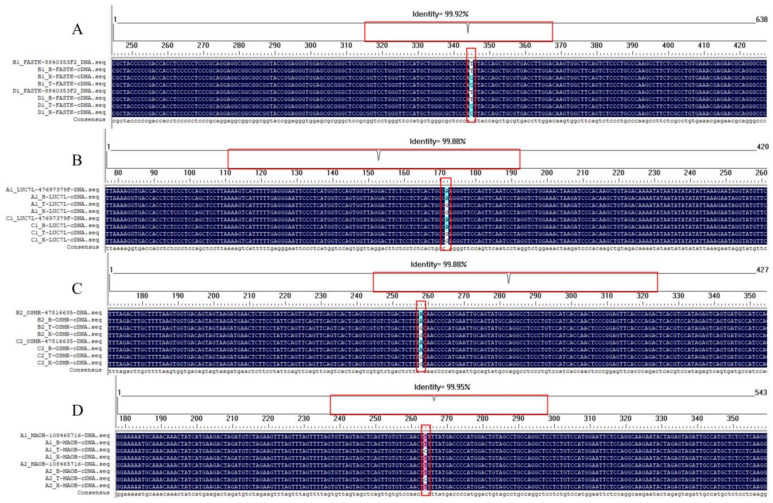
REs validation of filtering sites. (**A**–**D**) are the verification diagrams of the four genes FASTK, LUC7L, OSMR, and MAOB. Their mutations are all T-to-C and A-to-G mutations. The area circled in red represents the site.

After filtering REs by function and mutation type, 16 REs were selected for verification, 11 of which were successfully verified. The sequence information of the remaining sites is shown in Supplementary Materials Table S8. The genes located in these four sites are all related to RNA splicing, RNA metabolism, or muscle growth and development (Figure 7). Alignment of the DNA and cDNA sequences of the four selected REs revealed T-to-C and A-to-G mutations in all selected individuals.

## 4. Discussion

RNA editing is a post-transcriptional process that changes the nucleotide sequence of an mRNA transcript, altering the translated protein from what was encoded in the original DNA [36]. Many REs have been discovered in animals [37] and plants [38], and the number of studies on RE detection is increasing. However, there are no reports focused on how methionine affects muscle RNA editing. After filtering, we found 1116 RNA editing events, mainly consisting of T-to-C and A-to-G mutations, in three types of muscle tissue in Maiwa yaks. This is consistent with the results of a 2022 report by Shu [39] on animal production in plateau and plain environments. The RNA editing site distribution across the genome in Maiwa yak buttock muscles under feeding conditions was similar to our results.

We obtained high-risk REs with five known genes: *CD36*, *GPKOW*, *HNRNPLL*, *SMS*, and *TMOD4*. *CD36* is a protein-coding gene that encodes the fourth-largest glycoprotein on the platelet surface. It binds to long-chain fatty acids and promotes their transport into cells, thereby supporting muscle lipid utilization, fat energy storage, and fat absorption [40]. *CD36* is enriched in the ECM–receptor pathway, indicating that *CD36* is used in different tissues of yaks to promote muscle growth and development, and changes in this gene likely affect the normal development of yak muscle tissue. *GPKOW* is necessary for human pre-mRNA splicing [41]; however, there have been few studies on *GPKOW*. We speculate that *GPKOW* may affect mRNA splicing in Maiwa yak muscle tissue, thereby affecting RNA editing. *HNRNPLL* encodes hnRNPLL (heterogeneous ribonucleoprotein L-like protein) [42], which is an RNA-binding protein expressed in terminally differentiated lymphocytes. hnRNPLL regulates alternative pre-mRNA splicing and RNA stability [43] and is found in wheat. In Maiwa yak muscle tissue, *HNRNPLL*, similar to *GPKOW*, may affect RNA editing, but interactions between the two proteins are unclear. *TMOD4* is involved in actin filament organization, muscle contraction, and myofibril assembly. In 2023, a co-expression analysis of bovine muscle tissues [44] revealed that *TMOD4* was highly expressed and positively correlated with bovine skeletal muscle differentiation in an in vitro satellite cell differentiation model, indicating that *TMOD4* plays an important role in muscle development. This finding is similar to the results of the present study. Here, we identified other risk genes. Although the risk levels of these genes were not the highest, they indicate the biological significance of these genes for the growth and development of yak muscle, suggesting the need for further research.

A common RNA editing site in the three muscle tissues was linked to the same exon of the *MSRA* gene through the five_prime_utr. This gene encodes a ubiquitous and highly conserved protein that catalyzes the enzymatic reduction in methionine sulfoxide to methionine. In rats, immunolocalization and northern blot analysis of the protein revealed the presence of *MSRA* in nearly all tissues studied, albeit at different expression levels [45]. The earliest *MSRA* protein sequence was obtained from Bos taurus [46]. *MSRA* can regulate protein function by oxidizing or reducing methionine residues in proteins, as well as by repairing oxidative damage and restoring biological activity. Yaks live in plateau areas for a long time and can adapt to hypoxic and cold environments [47]. Our results show that RNA editing occurs in *MSRA*, identified by five_prime_utr. We speculate that because yaks live in low-oxygen areas for long periods of time, the addition of methionine to their basic diet can strengthen interactions between this gene and the environment, resulting in functional RNA edits.

We also detected a high-risk RE located at the X chromosome. It made the leucine mutate to proline, which is an important component of animal collagen. Comparing the sequence these REs with the human genome, it shared a 78.28% similar sequence with the *ELK1* gene. *ELK1* is a member of the T cell factor, or TCF, a subfamily of Ets transcription factors. Like other Ets transcription factors, it contains a conserved Ets domain [48]. The protein encoded by this gene is a nuclear target of the Ras-Raf-MAPK signaling cascade. This gene generates multiple isoforms via alternative translation initiation codons and splicing. Studies have found that *ELK1* plays an important role in the pathogenesis of various diseases, such as nasopharyngeal cancer [49] and pancreatic cancer [50]. We speculate that *ELK1* is used in yaks to regulate cell health in longissimus dorsi muscle tissues and gene expression in response to environmental conditions.

We analyzed the physical and chemical properties of the *ELK1* protein before and after mutation and found that the fat coefficient of the protein decreased after mutation. The aliphatic index, an index used to describe the amino acid composition of a protein, is an indicator of protein hydrophobicity and stability. A higher aliphatic index usually implies that a protein has greater hydrophobicity and stability. Aliphatic amino acids can form a hydrophobic core in the protein structure, thus helping to fold and stabilize a protein. After a mutation occurs at this site, the protein encoded by this gene becomes unstable, which may be detrimental to the development of muscle tissue in Maiwa yaks. This association requires further study.

The results of GO analysis showed that most of the gene-enriched entries were related to the development of muscle tissue, such as protein secretion, the establishment of protein localization to the extracellular region, protein localization to the extracellular region, the response to oxygen-containing compounds, and the response to lipids. Among them, muscle cells have a large demand for oxygen, and changes in oxygen content will trigger a series of adaptive responses. The signal transduction pathway of hypoxia-inducible factor 1 (*HIF-1*) and the *HIF-1α*/*mTOR* signaling pathway are key centers for many pathways that affect upstream milk protein factors and lipid synthesis-related genes [51]. According to the KEGG analysis, most of the detected genes were enriched in important pathways related to muscle development, such as ECM–receptor interactions, glycerophospholipid metabolism, thyroid hormone signaling, and insulin secretion. The ECM plays an important role in regulating cell proliferation, adipogenic differentiation, and preadipocyte migration [52]. Overall, our results indicate that RNA editing may affect muscle tissue development under the action of methionine.

## 5. Conclusions

During the addition of methionine to the yaks’ diet, an amount of RNA editing events were detected among muscle tissues. Some editions of high-risk sites, such as the MSRA gene, can regulate the reduction in methionine. These findings prove that RNA editing is an important response mechanism in organisms.

## Data Availability

Sequencing data for Maiwa yaks muscle tissue were deposited in the NCBI Sequence Read Archive under accession number PRJNA1199743.

## Supplementary Materials

The following supporting information can be downloaded at: https://www.mdpi.com/article/10.3390/ani15020171/s1. Table S1: Methionine supplementary feeding amount and percent; Table S2: Nutritional composition of feed; Table S3: Summary of RNA-seq data and mapping; Table S4: Summary of RNA editing sites in three muscle tissues detected using three software; Table S5: Number of variant types of REs predicted by at least two software in three tissues; Table S6: The risk level of REs; Table S7: *p*-values for KEGG pathways; Table S8: Primes of each validation of REs’ relative functional gene.
