# Supplementary material for: Identification of RNA Editing Sites Reveals Functional Modifications with the Addition of Methionine to the Daily Rations of Yaks
Source: Animals (Basel). 2025 Jan 10;15(2):171. doi: 10.3390/ani15020171 (PMC11758614; doi:10.3390/ani15020171)
Supplement: Supplementary file 1 [file animals-15-00171-s001.zip › animals-3360361-supplementary.pdf]

Table S1 Methionine supplementary feeding amount and percent

TAXA	CON	PRM1	PRM2	PRM3
Met additional (g/d)	\	5	10	15
Met detection (%)	0.06	0.19	0.32	0.44

24 Maiwa male yaks with similar weight (252.79 ± 15.95 kg) and good health condition of about 4 years old were selected as experimental animals. A completely randomized design was adopted. The samples were randomly divided into 4 groups and there are 6 yaks in each group as repetition. Respectively, the control group (CON, fed only the basic diet), the experimental group 1 (PRM1, supplemented with 5 g/d RP-Met), and the experimental group 2 (PRM2, supplemented with 10 g/d RP-Met) -Met), test group 3 (PRM3, supplementary feeding 15 g/d RP-Met), the CON group's dietary Met percent was 0.06%, the RPM1 group's dietary Met percent was 0.19%, and the RPM2 group's dietary Met percent was 0.32% , the dietary Met percent of the RPM3 group was 0.44%.

Table S2 Nutritional composition of feed

Component	
Diet composition	Content(DM,%)
Concentrated feed	
corn	15.75
Soybean	4.50
Soybean meal	7.65
Rapeseed meal	2.25
Corn Germ Meal	3.60
Spray corn husk	4.50
Bean curd	3.15
Molasses	2.25
Premix*	1.35
Roughage	
Corn stalk silage	55.00
Total	100
Nutritional Information	(DM,%)
Crude protein	13.13
Neutral detergent fiber	43.92
Acid detergent fiber	15.63
Organic matter	90.75
Calcium	0.95
Phosphorus	0.19

*:The premix provided per kg of diet: vitamin A 2 500 IU, vitamin D 550 IU, vitamin E 10 IU, copper 10 mg/kg, iron 50 mg, manganese 40 mg, zinc 40 mg, iodine 0.5 mg, selenium 0.2 mg, and cobalt 0.2 mg.

Table S3 Summary of RNA-seq data and mapping.

Taxa	Clean reads	Total mapped	Uniquely mapped
Longissimus dorsi	2,314,821,308	94.94%	92.43%
Buttock muscle	2,254,137,032	94.55%	91.98%
Pectorcl muscle	2,285,468,019	94.60%	91.98%

Table S4 Summary of RNA editing sites in three muscle tissues detected using three software

	Longissimus dorsi	Buttock muscle	Pectorcl muscle
JACUSA	12864	13037	13488
SPRINT	487	676	727
REDItools	52972	54031	55202

Table S5 Number of variant types of REs predicted by at least two software in 3 tissues

Type	Longissimus dorsi	Buttock muscle	Pectorcl muscle
T-to-C	112	139	165
A-to-G	84	130	135
C-to-T	26	32	29
G-to-A	18	16	16
C-to-G	12	10	11
A-to-C	11	10	12
A-to-T	11	11	12
G-to-C	10	10	10
G-to-T	10	8	10
C-to-A	9	7	7
T-to-G	7	6	3
T-to-A	5	5	5
G-to-C/G-to-T	1	0	0
C-to-G/C-to-T	0	1	0

Table S6 The risk level of REs

Taxa	Longissimus dorsi	Buttock muscle	Pectorcl muscle
High	12	13	12
Moderate	19	20	21
Low	9	9	12
Modifier	296	326	407

*High indicates a stop mutation or a frameshift mutation; Moderate indicates a missense mutation or structural deletion; Low indicates a synonymous mutation; Modifier indicates a non-coding mutation at a non-coding gene position or in a region downstream of the gene mutation.

Table S7 P values for KEGG pathways

	PATHWAYS	P.adjust	Gene
Longissimus dorsi	Nucleotide metabolism	0.00176	ENPP1/PNP/XDH
Longissimus dorsi	Nicotinate and nicotinamide metabolism	0.00537	KCNJ9/NAPEPLD/PRKACA
Longissimus dorsi	Purine metabolism	0.00567	ENPP1/PNP/XDH
Longissimus dorsi	Retrograde endocannabinoid signaling	0.0085	KCNJ9/NAPEPLD/PRKACA
Longissimus dorsi	Gastric acid secretion	0.01846	PRKACA/SLC4A2
Longissimus dorsi	ECM-receptor interaction	0.02482	CD36/ITGA1
Longissimus dorsi	Morphine addiction	0.0264	KCNJ9/PRKACA
Longissimus dorsi	Endocrine resistance	0.028	MDM2/PRKACA
Longissimus dorsi	Circadian entrainment	0.03137	KCNJ9/PRKACA
Longissimus dorsi	Salivary secretion	0.03194	PRKACA/SLC4A2
Longissimus dorsi	Glycerophospholipid metabolism	0.03547	AGPAT3/LPCAT2
Longissimus dorsi	Bile secretion	0.03607	PRKACA/SLC4A2
Longissimus dorsi	Dilated cardiomyopathy	0.03668	ITGA1/PRKACA
Longissimus dorsi	Hematopoietic cell lineage	0.03668	CD36/ITGA1
Longissimus dorsi	Serotonergic synapse	0.04426	KCNJ9/PRKACA
Buttock muscle	Necroptosis	0.00043	EIF2AK2/H2AC20/H2AC21/IFNAR1/IFNGR2
Buttock muscle	Alcoholism	0.00116	H2AC20/H2AC21/H2BC21/MAOB/PRKACA
Buttock muscle	Nucleotide metabolism	0.00344	ENPP1/PNP/XDH
Buttock muscle	Starch and sucrose metabolism	0.00545	AGL/ENPP1
Buttock muscle	Nicotinate and nicotinamide metabolism	0.00843	ENPP1/PNP

Buttock muscle	Serotonergic synapse	0.00856	KCNJ9/MAOB/PRKACA
Buttock muscle	Purine metabolism	0.01083	ENPP1/PNP/XDH
Buttock muscle	Dopaminergic synapse	0.0115	KCNJ9/MAOB/PRKACA
Buttock muscle	Cocaine addiction	0.01198	MAOB/PRKACA
Buttock muscle	Arginine and proline metabolism	0.01345	MAOB/SMS
Buttock muscle	Endocrine and other factor-regulated calcium reabsorption	0.01345	ATP1A2/PRKACA
Buttock muscle	Retrograde endocannabinoid signaling	0.01605	KCNJ9/NAPEPLD/PRKACA
Buttock muscle	Amphetamine addiction	0.02382	MAOB/PRKACA
Buttock muscle	Systemic lupus erythematosus	0.02781	H2AC20/H2AC21/H2BC21
Buttock muscle	Influenza A	0.03051	EIF2AK2/IFNAR1/IFNGR2
Pectorcl muscle	Gastric acid secretion	0.00208	ATP1A2/PRKACA/SLC4A2
Pectorcl muscle	Morphine addiction	0.00358	KCNJ9/PDE3A/PRKACA
Pectorcl muscle	Salivary secretion	0.00479	ATP1A2/PRKACA/SLC4A2
Pectorcl muscle	Bile secretion	0.00577	ATP1A2/PRKACA/SLC4A2
Pectorcl muscle	Thyroid hormone signaling pathway	0.00787	ATP1A2/MDM2/PRKACA
Pectorcl muscle	Nicotinate and nicotinamide metabolism	0.00795	ENPP1/PNP
Pectorcl muscle	Purine metabolism	0.00997	ENPP1/PDE3A/PNP
Pectorcl muscle	Endocrine and other factor-regulated calcium reabsorption	0.0127	ATP1A2/PRKACA
Pectorcl muscle	Retrograde endocannabinoid signaling	0.0148	KCNJ9/NAPEPLD/PRKACA
Pectorcl muscle	Renin secretion	0.02501	PDE3A/PRKACA
Pectorcl muscle	Thyroid hormone synthesis	0.02696	ATP1A2/PRKACA
Pectorcl muscle	Insulin secretion	0.03314	ATP1A2/PRKACA
Pectorcl muscle	Nucleotide metabolism	0.03532	ENPP1/PNP
Pectorcl muscle	ECM-receptor interaction	0.03605	CD36/ITGA1

Table S8 primes of each validation of REs relative functional gene

Gene	Sample		Sequence
ATP1A2		DNA	CTTTTGAAGGGATGACCCCCCTGCAAAATTC
ATP1A2	A1	Longissimus dorsi	CTTTTGAAGGGATGACCCCCCTGCAAAATTC
ATP1A2		Buttock muscle	CTTTTGAAGGGATGATCCCCCTGCAAAATTC
ATP1A2		Pectorcl muscle	CTTTTGAAGGGATGATCCCCCTGCAAAATTC
ATP1A2		DNA	CTTTTGAAGGGATGACCCCCCTGCAAAATTC
ATP1A2	B1	Longissimus dorsi	CTTTTGAAGGGATGATCCCCCTGCAAAATTC
ATP1A2		Buttock muscle	CTTTTGAAGGGATGACCCCCCTGCAAAATTC
ATP1A2		Pectorcl muscle	CTTTTGAAGGGATGACCCCCCTGCAAAATTC
GBP2		DNA	TGATCGCCCTGCTTATAGGAAGTACCTCA
GBP2	D1	Longissimus dorsi	TGATCGCCCTGCTTATAGGAAGTACCTCA
GBP2		Buttock muscle	TGATCGCCCTGCTTATAGGAAGTACCTCA
GBP2		Pectorcl muscle	TGATCGCCCTGCTTATAGGAAGTACCTCA
GBP2		DNA	TGATCGCCCTGCTTATAGGAAGTACCTCA
GBP2	D2	Longissimus dorsi	TGATCGCCCTGCTCATAGGAAGTACCTCA
GBP2		Buttock muscle	TGATCGCCCTGCTTATAGGAAGTACCTCA
GBP2		Pectorcl muscle	TGATCGCCCTGCTCATAGGAAGTACCTCA
SH3BP4		DNA	ACCCTTTGGAAACACAAAGGAGGTGATT
SH3BP4	A1	Longissimus dorsi	ACCCTTTGGAAACACAAAGGAGGTGATT
SH3BP4		Buttock muscle	ACCCTTTGGAAACACAAAGGAGGTGATT
SH3BP4		Pectorcl muscle	ACCCTTTGGAAACACAAAGGAGGTGATT
SH3BP4		DNA	ACCCTTTGGAAACACAAAGGAGGTGATT
SH3BP4	A2	Longissimus dorsi	ACCCTTTGGAAATACAAAGGAGGTGATT
SH3BP4		Buttock muscle	ACCCTTTGGAAATACAAAGGAGGTGATT
SH3BP4		Pectorcl muscle	ACCCTTTGGAAATACAAAGGAGGTGATT
NAPEPLD		DNA	TGTTTCTCTCTGCCCTTTGCCAGCATTATTTAG
NAPEPLD	A1	Longissimus dorsi	TGTTTCTCTCTGCCCTTTGCCAGCATTATTTAG
NAPEPLD		Buttock muscle	TGTTTCTCTCTGCCCTTTGCCAGCATTATTTAG
NAPEPLD		Pectorcl muscle	TGTTTCTCTCTGCCCCTTGCCAGCATTATTTAG
NAPEPLD		DNA	TGTTTCTCTCTGCCCTTTGCCAGCATTATTTAG
NAPEPLD	C2	Longissimus dorsi	TGTTTCTCTCTGCCCTTTGCCAGCATTATTTAG
NAPEPLD		Buttock muscle	TGTTTCTCTCTGCCCTTTGCCAGCATTATTTAG
NAPEPLD		Pectorcl muscle	TGTTTCTCTCTGCCCCTTGCCAGCATTATTTAG
DHX15		DNA	GCTGGTAATAGCCATGGCACTAGGGAAGA
DHX15	B1	Longissimus dorsi	GCTGGTAATAGCCATGGCACTAGGGAAGA
DHX15		Buttock muscle	GCTGGTAATAGCCATGGCACTAGGGAAGA
DHX15		Pectorcl muscle	GCTGGTAATAGCCATGGCACTAGGGAAGA
DHX15		DNA	GCTGGTAATAGCCATGGCACTAGGGAAGA
DHX15	B2	Longissimus dorsi	GCTGGTAATAGCCACGGCACTAGGGAAGA
DHX15		Buttock muscle	GCTGGTAATAGCCACGGCACTAGGGAAGA
DHX15		Pectorcl muscle	GCTGGTAATAGCCACGGCACTAGGGAAGA
CEMIP2		DNA	TTTGTGTGTGTGTTTGTTTTTTAATGA
CEMIP2	B2	Longissimus dorsi	TTTGTGTGTGTGTGTGTTTTTTAATGA

CEMIP2		Buttock muscle	TTTGTGTGTGTGTGTGTTTTTTAATGA
CEMIP2		Pectorcl muscle	TTTGTGTGTGTGTGTGTTTTTTAATGA
CEMIP2		DNA	TTTGTGTGTGTGTTTGTTTTTTAATGA
CEMIP2	C1	Longissimus dorsi	TTTGTGTGTGTGTTTGTTTTTTAATGA
CEMIP2		Buttock muscle	TTTGTGTGTGTGTTTGTTTTTTAATGA
CEMIP2		Pectorcl muscle	TTTGTGTGTGTGTTTGTTTTTTAATGA
MSRA		DNA	CCGACCGCCCGGCGGCCCCTTGGCCAG
MSRA	A1	Longissimus dorsi	CCGACCGCCCGGCGGCCCCTTGGCCAG
MSRA		Buttock muscle	CCGACCGCCCGGCGGCCCCTTGGCCAG
MSRA		Pectorcl muscle	CCGACCGCCCGGCGGCCCCTTGGCCAG
MSRA		DNA	CCGACCGCCCGGCGGCCCCTTGGCCAG
MSRA	A2	Longissimus dorsi	CCGACCGCCCGGAGGCCCCTTGGCCAG
MSRA		Buttock muscle	CCGACCGCCCGGAGGCCCCTTGGCCAG
MSRA		Pectorcl muscle	CCGACCGCCCGGAGGCCCCTTGGCCAG
